# Supplementary material for: Are carbon emissions trading and green financial instruments synergistic? -Comprehensive quantitative research based on content analysis
Source: PLoS One. 2024 Mar 7;19(3):e0298601. doi: 10.1371/journal.pone.0298601 (PMC10919601; doi:10.1371/journal.pone.0298601)
Supplement: S1 Data — (ZIP) [file pone.0298601.s001.zip › Data/地级市Z_F工作报告.zip!/╣π╢1⁄2/╔╟╬▓╩╨/└┤╫╘╣1⁄2╓┌║┼í╛┬φ┐╦╩2╛▌═°í┐_2011.docx]

各位代表：

　　我代表市人民政府向大会作政府工作报告，请予审议，并请政协各位委员和其他列席人员提出意见。

　　2010年及"十一五"时期工作回顾

　　2010年，市政府在党中央、国务院和省委、省政府及市委的正确领导下，在市人大、市政协的监督、支持下，深入贯彻落实科学发展观，认真贯彻落实党的十七届五中全会、省委十届六次、七次全会和汪洋书记、黄华华省长有关重要讲话精神，以及市委五届七次、八次全会精神，以"四推进一保持"为工作主线，以"五年大变化"和全面砍掉落后"尾巴"为目标，以产业集聚力促现代产业体系，以宜居城乡力促扩大内需，以"三大机制"力促工作落实，巩固和发展了来之不易的大好局面，经济社会持续较快发展，"活力汕尾、人文汕尾、和谐汕尾、清新汕尾"建设加快推进。概括起来，主要体现在以下九个方面：

　　一、综合实力跨上新台阶

　　全年预计，全市实现地区生产总值470亿元，比上年增长18.1%；工业增加值186亿元，增长25.5%；规模以上工业增加值104亿元，增长32%；全社会固定资产投资364.6亿元，增长26%；实际吸收外商直接投资2.84亿美元，增长3%；外贸出口总值11亿美元，增长16%；财政一般预算收入26.2亿元，增长33.1%；社会消费品零售总额341.6亿元，增长21.5%；农业总产值129.1亿元，增长6.5%；市区居民人均可支配收入13850元， 增长10.3%；农民人均纯收入6000元，增长11.8%。三次产业结构由2009年的17.3∶44.5∶38.2调整为16.7∶46.5∶36.8。

　　二、重点项目建设取得新进展

　　2010年列入省、市重点建设项目54个，预计全年完成投资148.8亿元，完成年度计划的106%。其中，进度较快的有汕尾电厂一期3#、4#机组、厦深铁路（汕尾段）、5座水库除险加固工程、城镇污水处理设施、汕尾市普及高中阶段教育工程、华师附中汕尾学校、市区碧桂园商住房一期工程、汕遮公路改（续）建工程等。新增中央投资项目四批共73个，开工率100%。全市共争取各类建设项目55个，得到中央和省专项补助资金3.9亿元，带动地方投资15.3亿元。华润火电、陆丰核电获得国家批准开展前期工作"路条"。

　　三、转变方式实现新突破

　　转型升级成效显著。自主创新能力不断增强，1-11月份全市专利申请量267件，增长9%；专利授权量240件，增长94%，发明专利授权量增幅继续居全省第一。信利4.5代AM-OLED、雅芝TFT、五丰水产、海崇畜牧和生宝种养等5个项目列入省现代产业500强项目。引进天洋太阳能光伏、"云计算"汕尾研究院、科亮特太阳能灯等新项目，电子信息等高端产业集聚明显加快。毛纺业数控化水平大幅提高，全市数控纺织机达4000多台。纺织服装、食品制造、金银珠宝首饰和圣诞礼品等支柱产业和产业集群加快发展。

　　工业经济稳步发展。开展"工业年"活动成效显著，预计全年全市实现工业总产值725亿元，增长25%。规模以上工业总产值增长40%，规模以上工业增加值增长32%，两项指标均在全省排名第5位。新增规模以上工业企业120家。全年全市工业用电量12.03亿千瓦时，增长19.9%，增幅居全省前列。工业经济效益明显提升，1-11月份，规模以上工业利润总额11.9亿元，增长21.5%。规模以上工业产值占工业总产值比重进一步提升，达到59%。工业对经济增长的贡献率达57.3%，拉动经济增长10.4个百分点。节能降耗取得明显成效，预计2010年万元GDP能耗下降1.5%，顺利完成年度及"十一五"节能目标。

　　"三农"工作扎实推进。粮食生产喜获丰收，全年粮食总产量51.5万吨，增长15.5%，超额完成省粮食工作考评指标。水产品总产量56万吨，增长2%；渔业经济总产值65.4亿元，增长5.3%。"十大农业种养基地"和"五大园区"建设初见成效，农业基地化、规模化、产业化、现代化进程加快。建成汕尾市现代农业示范区、东兴喷灌种植基地、陆港无公害蔬菜生产基地、海丰联安有机米基地等一批标准较高的农业生产园区和基地。全市新增市龙头企业13家。"十大标准化渔港"建设顺利推进，汕尾渔港被评为全国首批"文明渔港"。水利工程建设稳步推进，全年完成投资2.3亿元。完成了25宗防灾减灾、54宗小型水库除险加固和102万人的农村饮水安全工程。造林绿化工作扎实推进，森林覆盖率达到47%，海丰县创建林业生态县通过省的验收。

　　第三产业增效明显。预计第三产业完成增加值173亿元，增长13.8%。消费市场持续畅旺，商贸旅游日显繁荣，房地产业、交通运输、邮电通信业快速发展。全年新增个体工商户1.2万户，增长120%；新增私营企业1275家，增长316%。"家电下乡"活动成效明显，"万村千乡"市场工程建设加快。全年接待游客341.8万人次，增长41.2%；旅游总收入35.2亿元，增长40%。

　　四、财税工作凸显新成效

　　财政收入继续保持大幅增长，全市完成地方财政一般预算收入26.2亿元，增长33.1%，增幅居全省第4位，其中：税收收入完成16.8亿元，增长34.1%，增幅居全省第3位，高出财政一般预算收入1个百分点，非税比略有下降。财政收支平衡，略有节余。金融形势稳定，全市年末本外币各项存款余额332.70亿元，比年初增长18.91%；本外币各项贷款余额131.12亿元，比年初增长33.02%。

　　五、深汕合作形成新亮点

　　深汕特别合作区得到省委、省政府和深圳市委、市政府高度重视，以"创新发展，先行先试，创建全省区域合作创新示范区"为目标，深汕两市密切、务实、积极合作，创新行政、财政、投入、管理机制，以汕尾所有、深圳所用，汕尾管辖、深圳开发，汕尾发展、深圳拓展，汕尾空间、深圳形象的全新模式，得到省的高度赞许和社会的高度关注，将上报省委、省政府争取批准为省级发展战略。作为合作区首期的深圳（汕尾）产业转移工业园基础设施逐步完善，已建成11栋标准厂房、排洪渠、1号路等基础设施。招商引资成效显著，深汕产业转移招商会上签约项目49个，投资总额533亿元，已引进恒兴水产、艾特航模、灏芯微电子、科亮特太阳能灯等一批重大项目落户合作区。

　　六、宜居建设开创新局面

　　2010年底规划编制工作基本完成，全面实现了"四规合一"，为建设宜居城乡提供了重要保障。重大市政工程项目建设快速推进，金湖路、腾飞路东段、成业路南段、大马路、三马路等市政道路已基本完工。汕遮公路全线通车。市区电网建设加紧推进。红海湾亚运赛区及市区市容市貌整治成效显著。陆河水质净化厂和海丰县污水处理厂已建成投产。垃圾处理项目已完成前期工作。城市管道天然气项目已逐步在市区点火供气。社会主义新农村建设稳步推进，全市有162个村庄开展了整治工作，有48个村已完成村庄规划，陆河县螺溪镇、新田镇丰山村分别被确定为全省首批宜居城镇创建指导点和宜居村庄创建指导点。

　　七、对外开放取得新进步

　　预计全年全市进出口总额20亿美元，增长14%，其中，出口11亿美元，增长16%，完成指标超过金融危机前的水平，实现恢复性增长。预计全市实际吸收外商直接投资2.84亿美元，增长3%。海丰小漠港、陆丰甲湖湾新增列入广东省"十二五"口岸发展规划，口岸全方位开放格局初步形成。招商引资取得新进展，全年签约、开工、竣工项目共230多个，投资总额达1400多亿元，为我市开放型经济发展注入了强劲活力。

　　八、社会事业再创新佳绩

　　涉及人民群众切身利益各项问题得到较好解决。社会保险覆盖面不断扩大，全市企业养老保险覆盖率由2009年的35%增长到53%，养老、失业、工伤、生育保险实行了市级统筹，新型农村养老保险试点顺利推进，陆河县试点45岁以上重点人群参保率达到88%，其中60岁以上老人参保率达到100%。全市征收五项社保费7.1亿元，增长20.5%，全市3万多名企业离退休人员养老金等各项社保待遇按时足额发放。全年全市新增城镇就业4.2万人，转移农村劳动力6万多人，城镇登记失业率为2.7%。保障性住房建设不断推进，解决了337户低收入家庭的住房困难。城乡社会救助体系不断完善。低保救助实现提标进位。五保供养标准不断提高。扶贫"双到"工作成效明显，全市已有34.2%贫困户实现脱贫。教育工作实现重大突破，小学学龄儿童入学率99.5%，特殊教育入学率93.5%，初中毕业生升学率91.2%，高中毛入学率83%（预测），增幅居全省前列。华师附中汕尾学校完成首期建设并招生，陆河教育园区交付使用。办学条件明显改善，教育教学质量稳步提高，2010年高考入围率83.5%，连续六年超过省平均线。人口计生工作取得新成绩，人口出生率、自增率分别是10.46&permil;和5.90&permil;，同比下降0.11和0.16个千分点，计划生育率95.87%，同比上升2.12个百分点，陆丰有望甩掉计生尾巴。文化、体育事业加快发展，文化设施建设加快推进，"农家书屋"、"村村通"等文化惠民工程加快建设，成功举办亚运会火炬传递文艺表演、"扶贫济困助教，共筑和谐家园"大型文艺晚会等活动，丰富了广大人民群众的文化生活；成功举办第14届亚洲帆船锦标赛和协办第16届亚运会帆船赛，得到省委、省政府和亚组委的嘉奖。医药卫生体制改革稳步推进，重大传染病和突发公共卫生事件得到有效防控，新型城乡医疗卫生服务体系进一步完善，新农合参合率达99.81%。环保工作取得新突破，实现了治污减排"砍尾"目标。产品质量和食品安全监督管理不断强化。成功摘掉陆丰金驿市场省"重点打假市场"和市城区香洲街道省"重点打假区域"帽子。打击非法传销成效显著，连续六年保持"零发案"。社会局面平安稳定，刑事立案数、每万人刑事发案数均排在全省末位，亚运会等国家重大活动期间没有发生进京到省集体上访和非正常上访，亚运会红海湾赛区全赛期实现"零安全事故、零刑事发案、零非正常上访、零群体事件、零投诉"的"五零"目标。安全生产"四项指标"全面下降，总体测评优良。精神文明建设扎实开展，社会文明程度不断提高。统计、审计、国土、法制、人事、国防、打私、气象、防震、台务、物价、林纸、司法、港澳、老龄、残联、民族宗教、外事侨务、档案修志、妇女儿童、工会、青年等工作取得新成绩。

　　九、民主法制和政府自身建设迈出新步伐

　　坚持依法治市，认真执行人大及其常委会决议，重视发挥人民政协的政治协商和参政议政作用，自觉接受人大依法监督、政协民主监督、舆论监督和公众监督，积极办理人大代表议案和政协委员提案。主动加强与各民主党派、工商联及人民团体的联系，广泛听取社会各界人士的意见，促进决策的科学化、民主化和法制化。全面推进依法行政，"权威、责任、效能、廉洁"政府建设稳步推进，积极推行行政执法责任制，规范行政执法行为，强化行政执法监督，政府工作法制水平不断提高。坚持用"责任、督查、奖罚"三大机制推动重点项目和重要工作的落实，领导干部执行力不断增强。深入开展"行风热线"、"民主评议公务"活动，加强对各级机关作风的明查暗访，政府自身建设不断加强，机关作风、办事效率和服务水平进一步提高。

　　在大家的共同努力下，2010年的工作计划顺利完成，为全面完成"十一五"规划打上了一个完美的句号。

　　各位代表，"十一五"时期是汕尾发展历程中极不平凡的五年。五年来，全市上下积极应对自然灾害、国际金融危机等多重不利因素和兄弟市加快发展、万马奔腾局面的影响和挑战，攻坚克难，开拓创新，"十一五"规划主要目标任务圆满完成，"砍尾"目标基本实现，为"十二五"发展奠定了坚实基础。

　　综合实力显著增强。全市生产总值从2005年的205.7亿元增加到2010年的470亿元（预计数，下同），年均增长16.9%，翻了一番多；人均生产总值由2005年的7419元提高到2010年的15932元（折合2400美元），年均增长15.4%，也翻了一番多；财政一般预算收入由2005年的7.09亿元增至2010年的26.23亿元，年均增长29.9%，接近翻两番。

　　三次产业协调发展。农业基础地位进一步巩固。工业增加值从2005年的70.49亿元增至2010年的186亿元，年均增长23%。商贸旅游业等第三产业不断壮大，社会消费品零售总额从2005年的130亿元增至2010年的341.6亿元，年均增长21.3%。三次产业结构由2005年的24∶39.7∶36.3调整到2010年的16.7∶46.5∶36.8。

　　基础设施日臻完善。2006-2010年，全市累计完成固定资产投资1174亿元。新（改）建公路1685公里，完成农村硬底化公路1745公里；汕尾电厂一期1#、2#机组建成并网发电，3#机组已建成试运行。全市形成了比较完善通畅的交通、通信、供水网络和安全运转的水利、供电网络。城镇化水平达到58.6%，中心市区面积也从建市初的4平方公里扩展为21平方公里。汕尾已逐步发展成为初具规模、充满活力的美丽滨海城市。

　　生态建设成效明显。"五大生态安全工程"建设扎实推进，节能减排力度不断加大，环境质量明显改善。预计"十一五"期间全市万元GDP能耗下降10%，圆满完成节能任务。二氧化硫排放量和化学需氧量排放量分别达到省下达的控制目标。

　　社会民生不断改善。基本公共服务逐年改善，教育、卫生、文化事业不断进步，社会保障体系逐步健全。人民生活明显改善，幸福指数不断提高。"十一五"期间建设保障性住房1270套。高中阶段教育毛入学率从2005年的35.36%增至2010年的83%。农民人均纯收入由2005年的3843元增至2010年的6000元，年均增长9.3%，城镇居民人均可支配收入由2005年的8311元增至2010年的13850元，年均增长10.8%。

　　改革开放取得突破。一些重要领域和关键环节改革不断深化，政府职能不断转变。对外开放水平稳步提高，外向型经济稳步发展，一批重大项目相继落户，五年累计全市吸收外商直接投资11.63亿美元。外贸出口总额由2005年的6.42亿美元增至2010年的11亿美元，年均增长11.4%。

　　民主法治和精神文明建设得到加强。民主法治建设深入推进，普法工作和民主评议行风、政风工作扎实开展，依法行政水平不断提高。"十一五"期间，我市被评为中国民间文化艺术之乡、最喜爱的休闲城市、全省双拥模范城。陆河县被评为中国青梅之乡、中国建筑装饰之乡、中国农村水电之乡。

细数"十一五"，我市发展速度持续高于全省，各项落后尾巴基本砍掉，区域协调发展抓住新机遇，历史遗留问题逐步解决，汕尾形象得到全新树立，人民群众得到更多实惠。可以说，"十一五"期间，是汕尾加快发展、迎头赶上的五年，是抢抓机遇、激发活力的五年，是树立形象、重振魅力的五年，是凝聚民心、团结奋进的五年。

各位代表，2010年及"十一五"期间取得的成绩来之不易，是在省委、省政府和市委的正确领导下，在市人大及其常委会、市政协的大力支持下，全市人民共克时艰、团结拼搏的结果。在此，我谨代表市人民政府，向全市广大工人、农民、知识分子、干部职工，向驻汕人民解放军、武警官兵和各民主党派、人民团体、社会各界人士表示崇高的敬意！向所有关心支持我市发展的港澳同胞、台湾同胞、海外侨胞、国际友人以及外地朋友表示衷心的感谢！

　　回眸2010年和"十一五"的工作， 我们得到许多有益启示：一是必须以科学发展观为指导，牢牢把握科学发展、跨越发展主题，加快转变经济发展方式，不断优化发展环境，打造新的发展平台。二是必须不断解放思想，坚持改革开放，与时俱进，以思想大解放促进经济社会大发展。三是必须立足大视野、规划大经济、谋划大发展，突出抓好招商引资工作，大力推进重点项目建设，努力推进经济新飞跃。四是必须强化发展氛围，团结广大干部群众，齐心协力，一心一意谋发展，形成加快发展的强大合力。

　　在充分肯定成绩的同时，我们也清醒地认识到，我市经济社会发展仍存在一些问题和困难，主要是：经济总量小、规模小，工业增长拉力不够强大，规模以上工业增加值仍然是尾巴；产业集聚水平仍然较低，工业园区发挥的集聚作用仍然不强，产业转型升级压力仍然巨大；经济效益不高，财力薄弱，经济运行质量效益依然处于低位；软环境有待不断改善，干部的执行力有待提高，政务环境还须下大功夫优化；教育、计生、社会保障和社会治安等压力依然巨大。对此，我们必须采取有效措施加以解决。

　　"十二五"时期发展目标和工作重点

　　"十二五"时期，是我市全面建设小康社会、转变经济发展方式的攻坚时期，是实现"五年大变化"、"十年大发展"的关键时期，也是我市实现科学发展、谋求后来居上的黄金时期。综观"十二五"时期国内外发展大势，汕尾既面临难得的发展机遇，又面临严峻的挑战，但总体上机遇大于挑战。当前，世界经济逐步复苏，总体有利于改善我市发展的外部环境。国家、省部署新一轮发展战略，总体有利于我市拓展发展空间。珠三角一体化、粤东西北区域协调发展进程加快和高铁时代的综合效应，我市将成为连接京广铁路、京九铁路、东南沿海铁路等三大铁路通道的交汇点和重要节点，成为区域交通枢纽城市，全面融入珠三角，有望成为"珠江东岸通衢"。随着深汕合作的深度推进，一批重大项目的落户、上马，将引领汕尾进入发展的黄金时期。与此同时，世界经济中不稳定不确定因素依然存在，国内各地竞相加快发展，竞争异常激烈；我市产业层次总体偏低，产业优化任务艰巨；改革的复杂性和艰巨性明显增大，社会管理的压力进一步加大；全面建设小康社会关键时期的各项任务对属于后发地区的我市必将形成现实考验。对此，我们必须增强忧患意识、使命意识和责任意识，切实抓住和利用好当前所面临的历史性机遇，审时度势，趋利避害，乘势而上，努力实现全市经济社会又好又快发展。

　　"十二五"时期我市经济社会发展的预期目标是：全市生产总值年均增长17%，2015年达到1000亿元以上，人均GDP达到3.4万元左右（约折合5000美元）；产业结构和产业布局明显优化，第三产业比重达到39%；地方财政一般预算收入超过70亿元，年均增长23%以上；重点经济区域形成重要拉动引擎，经济发展方式明显转变，资源利用效率显著提高，清新汕尾建设取得成效，宜居城乡生态环境明显改善。节能减排力度进一步加大，全面完成省下达目标。交通基础设施更加完善，社会保障体系逐步健全，城乡居民收入明显提高，社会繁荣和谐，人民安居乐业，人民生活总体上达到较为宽裕的小康水平。

　　各位代表，未来五年，是汕尾科学发展、跨越发展的关键时期。我们一定要以强烈的使命感和责任感，全力推进各项工作，科学跨越发展，建设幸福汕尾，努力把"十二五"宏伟蓝图变成美好现实。

　　2011年工作安排

　　2011年是实施"十二五"规划的开局之年，是转变方式的关键之年，也是实现"五年大变化"目标的决战年。

　　今年政府工作的总体要求是：高举中国特色社会主义伟大旗帜，坚持以邓小平理论和"三个代表"重要思想为指导，以科学发展观统揽全局，全面贯彻党的十七届四中、五中全会、中央经济工作会议精神和省委十届八次全会及市委五届九次全会精神，以实现"五年大变化"和全面"砍掉落后尾巴"为目标，以"科学发展、跨越发展"为主题，以"转变方式、共享和谐"为主线，深入实施"深汕合作、海岸经济、宜居城乡、民生为本"四大战略，推进科学跨越发展，建设幸福汕尾。致力建设电力能源、电子信息、石油化工、旅游休闲、现代农业、现代物流六大基地，加快融入珠三角，持续推进经济社会又好又快发展，打造"活力、人文、和谐、清新"的宜居宜业宜憩的现代化滨海城市。

　　今年经济社会发展的预期目标是：地区生产总值增长18%；农业总产值增长6.5%；规模以上工业总产值增长40%，规模以上工业增加值增长35%；全社会固定资产投资增长28%，社会消费品零售总额增长18%，外贸出口总额增长18%；外商直接投资增长20%；地方财政一般预算收入增长28%；城镇居民人均可支配收入增长12%，农民人均纯收入增长11%；居民消费价格涨幅控制在4%左右；城镇登记失业率控制在3%以内；人口自然增长率控制在6.5&permil;以下；单位生产总值能耗下降、化学需氧量排放量减少、二氧化硫排放量减少，完成省下达的指标。

　　为实现今年经济社会发展预期目标，政府工作要以落实、扩展、优化、提高为工作主线，重点抓好"十个强力推进"：

　　一、强力推进重点项目建设，增强经济发展实力

　　推进重点项目建设。2011年计划安排重点项目36个，总投资514亿元，年度计划投资66亿元。涉及交通通信、工业物流、农林水利、城市基础设施、社会事业和民生保障等领域。确保汕尾电厂3#、4#机组建成发电，重点抓好陆丰核电、华润海丰电厂、垃圾处理场（厂）、品清湖疏浚整治等重点项目开工建设。

　　加快交通大网络建设。按照打造粤东通向珠三角桥头堡的新定位，加快推进"两网一港"（公路网、铁路网、海港）建设。要加快推进厦深铁路（汕尾段）及配套站场建设，全力抓好广汕高铁、潮惠高速、海汕公路埔边北段、国道324线（汕尾段）改造、绿道、东涌火车站、市区银龙湾隧道、汕尾新港等交通项目开工建设或完成前期工作。

　　推进民心工程建设。继续实施民心工程，完成自然村广播电视村村通建设，实现100%行政村接入互联网，普及率达到60%。加快公共基础设施建设，解决饮水安全问题。大力实施城镇安居工程、农村安居工程，2011年计划建设保障性住房350套。

　　二、强力推进现代产业体系建设，加大力度转变发展方式

　　增强自主创新能力。加大科技投入，大力扶持企业为主体的自主创新体系建设，引进项目研发中心、研究院所、产业孵化器，重点利用"云计算"、灏芯微电子、天洋太阳能光伏等高端技术项目带动研发基地落户，创建省级高新技术开发区，加强与中国科学院合作的"院地合作"联盟，深化产学研合作，逐步提升科技对经济发展的贡献水平。

　　做大规模以上工业。引进投资规模大、发展前景好、带动能力强的大项目，支持本地企业与大型企业集团"联姻"，扶持有潜力的企业做大做强，形成一批规模以上工业企业骨干。落实扶持民营经济发展的政策措施，促使更多民营企业上规模上档次。落实领导抓工业责任制，规范工业企业管理，加大工业企业清理清查力度，推进规模工业上新台阶。

　　集聚发展电子信息产业。继续加快信利、德昌等龙头企业的发展，做大提升电子信息这一优势产业，培育物联网、云计算、三网融合、网络增值服务等新兴产业，加快信利4.5代AM-OLED、雅芝TFT、天洋太阳能光伏、"云计算"、 灏芯微电子、科亮特太阳能灯等项目建设，加快承接珠三角东岸电子信息业的转移、对接，形成具有较强竞争力的高端新型电子信息产业集聚区。

　　优化发展电力能源产业。加快推动华润海丰电厂等临港火电项目建设，开工建设6台108万千瓦机组的陆丰核电站，优化发展风电、太阳能发电等新能源项目。做好电网科学规划，加快电网建设步伐，确保电力项目及时并网发电投入商业运行。

　　精细发展石油化工产业。充分发挥我市良好的区位优势，主动承接周边炼化项目的下游产业，以中海油华城石化储运项目、粤东成品油管道（汕尾段）建设为起点，重点发展面向粤东、珠三角和闽东南市场的石油储运、精细化工，为珠三角石化产业带向东延伸提供腹地。

　　不断提升传统产业。实施名牌带动战略，加大投资力度，做大做强纺织服装、食品制造、金银珠宝首饰加工、圣诞工艺品等传统产业集群。依法关闭高污染、高能耗、低效益的企业。推进自主创新，扶持发展一批拥有自主知识产权的名牌产品、驰名商标，力争传统支柱产业依托名牌、技改升级转型。

　　三、强力推进宜居城市建设，加快城市化进程

　　加快中心城区建设。全面加快宜居城乡规划建设，以"海在城中、城在山间"为主线，打造宜居、宜业、宜憩三大功能，努力创建国家宜居示范城。围绕打造"一（中）心一轴、一带三片（区）、多组团"的城市发展空间结构，推动汕尾市区适度东拓，建设汕尾新中轴线。全力推进奎山河、品清湖的综合整治和百里海堤工程建设。加强城市管理，重点治理脏、乱、差现象，提升城市居民幸福感、荣誉感。

　　完善市区基础设施。全力推进市区规划中轴线新湖大道、工业大道、环品清湖道路和市区绿道网建设。加快市区电网建设，力争110千伏品清、马宫输变电工程建成投产。加快中国（汕尾）民间艺术馆、市体育中心、市规划博物馆等公益性基础设施建设。继续抓好红海西路、市区东区污水处理厂管网工程和市区西区污水管网改造工程建设。

　　推进房地产业健康发展。认真贯彻落实国家和省有关房地产业方针政策，增加面向中低收入家庭的中低价位、中小套型普通商品房供给，改善市场供求关系，稳步推进市区房地产业合理的发展规模和增长水平。大力推进市区碧桂园二期、金海华府、银湖湾等大型房地产建设。抓好和顺村、小岛村、渔村的"三旧"改造，改善优化生活居住环境。

　　保护城市生态环境。强化造林绿化、水土保护，加快公共绿地建设，推动生态文明城、镇、村建设。开建"千村千园"（村头公园）、"千里绿道"和百里海堤绿化工程，力争三年到位。大力发展循环经济，推行清洁生产；落实节能措施，确保节能工作合格；严格环保市场准入，从源头上控制污染；继续抓好"生态安全工程"建设，重点抓好海堤、减排、治污、绿化、改水等五大工程的扫尾工作；严格控制建设用地规模，提高土地利用效率；大力发展低碳经济。

　　四、强力推进深汕特别合作区建设，打造经济发展新引擎

　　举全市之力推进深汕合作。以汪洋书记去年8月25日在汕尾调研时重要讲话精神为动力，举全市之力，全面推进深汕特别合作区建设。积极争取省的大力支持，以最新机制、最优政策、最好服务、最高质量、最美环境、最佳效益的高标准建设园区，以"创新发展，先行先试"为核心，努力创建全省区域合作创新示范区；主动加强与深圳的深度对接，运用深圳的品牌和经验，用好用足资金投入、项目支持、土地利用等各项支持政策，全方位推进合作区发展，实现创新合作首年的制度创新全面到位、基础设施全面展开、招商项目全面上马。

　　做强合作区产业聚集效应。高起点建设投资创业载体，吸引更多内外资企业前来投资，大力发展技术密集型、资金密集型和外向型产业，努力培育产值10亿元级以上的项目。积极跟踪已签约项目，提高项目履约率，特别要争取"云计算"、灏芯微电子、雅芝TFT、天洋太阳能光伏、恒兴水产等项目早日上马，争取科亮特太阳能灯、艾特航模、凌祺电子设备、华阳通信设备等项目早日投产。同时，加强与珠三角地区行业组织的联系，吸引珠三角地区产业组团式转移入园。

　　及早做好全市接受合作区辐射的准备。按照"近期建园，中期为区，远期为城"的发展思路，合作区将把深圳对外辐射能力向我市拉近100多公里，来到家门口。如何对接合作区，引领辐射力，提升融合力，使到合作区真正对全市产业发展和转变方式起到直接、重大、良好的影响，将是考验我们决断力、执行力的全新课题。必须从现在起就加强完善各项基础设施，加快建设功能完善的对接平台，实施与路网、站点、港区、仓储的全面对接，加大其他开发区的功能配套、完善力度，使合作区的功能得到延伸和拓展。

　　五、强力推进招商引资工作，提升对外开放水平

　　进一步优化招商环境。着力在优化服务上下功夫，继续完善"金钥匙保姆式服务"、"一站式行政服务"、"订单式服务"， 进一步健全县（市、区）招商引资责任制和外经贸联席会议制度，努力营造开明开放的招商环境、高效运转的体制环境、维权护商的服务环境、安宁宽松的社会环境和诚实守信的信用环境，形成"洼地效应"，吸引集聚型投资。

　　进一步加大招商引资力度。以园区招商和产业招商为切入点，加强对外招商宣传推介，丰富合作方式，拓宽招商渠道，提高利用外资的规模和质量。积极组织参加省组织的各类招商会，加大引资力度。积极引导外资投向主导产业、高新技术产业、现代服务业和节能环保产业等领域。吸引世界500强、中国500强企业、中央企业和大型民营企业到我市投资。落实项目责任，力促招商项目尽快履约、建设、投产，早出效益。

　　进一步抓好外贸出口。切实落实外贸出口扶持政策，促进企业扩大出口。加快转变外贸发展方式，积极培育以技术、品牌、质量、服务为核心竞争力的新优势，推动加工贸易企业延长产业链和增值链，提高出口产品档次、附加值和竞争力。实行出口市场多元化，促进出口结构转型升级。积极培育和壮大出口主体，帮助民营企业申报进出口经营权，推进企业加大国际市场开拓力度，增强外贸出口实力和发展后劲。积极推进文明口岸建设，继续营造"大通关"良好环境。

　　六、强力推进消费需求，促进第三产业快速发展

　　大力发展港口物流业。优化岸线布局，努力形成港城互动的良好局面。加快汕尾港物流园区、深圳（汕尾）产业转移园物流园区、汕尾火车站物流园区、陆丰内湖农副产品物流中心、海丰县中宝珠宝交易物流中心建设。谋划建设汕尾新港区，把汕尾新港区规划成珠三角地区新的港口物流基地，力争建成国家一级一类的国际港口。引导现有运输、仓储、代理等企业进行服务延伸和功能整合，大力发展第三方专业化物流。

　　大力发展滨海旅游业。修编旅游发展规划，整合"红、蓝、绿、古、特"五色资源，构建不同风格的滨海、生态和城市景观区。加大旅游宣传促销力度，重点承接珠三角东岸重要客源市场。全力推动内湖墟二期、长沙湾度假城、明热温泉、好日子大酒店、黄金海岸大酒店等旅游项目上马开工建设，把汕尾打造成珠三角东大门的休闲度假胜地和现代旅游新城。

　　大力发展现代服务业。坚持把发展现代服务业作为构建现代产业体系的战略重点，加快构筑与珠三角地区服务业有机接轨的现代服务业体系。优先发展为工业配套的金融服务、电子商务、信息咨询、技术研发等生产性服务业。把市区建设成集购物、休闲、餐饮和娱乐等功能于一体的核心商贸区。

　　大力促进消费需求。落实好国务院搞活流通扩大消费相关措施，积极拓宽消费领域，增强消费对经济增长的拉动作用。努力扩大住房、汽车等大众消费，大力发展社区商业、物业、家政等服务性消费，积极发展文化娱乐、休闲旅游、体育健身等热点消费。深入实施"万村千乡"市场工程、"家电下乡"工程，培育农村新的消费热点。

　　七、强力推进"三农"工作，加快社会主义新农村建设

　　大力改善农村生产生活条件。加强城乡规划，结合"三旧"改造，全面推进村庄整治建设，从2011年起每年完成10%自然村整治建设任务，促进农村人居环境和村容村貌实现根本改观。大力推进防灾减灾、病险水库除险加固、农村饮水安全等水利工程建设，加快发展水利经济。

　　加快发展效益农业、生态农业。加大农业招商引资力度，大力发展高效、优质、生态的现代农业，建设珠三角地区农产品生产供应基地。加快农业和农村经济结构调整，构建特色农业产业体系。继续打造水稻、蔬菜、水果、薯类、花生、油茶、花卉苗木、生猪、黄牛、肉鸡等十大现代农业基地。启动实施原生态农产品工程，加快实施农业品牌战略，创建一批农业名牌产品。

　　大力发展海洋水产业。把握我省被列为全国海洋经济发展试点省的契机，加快海洋综合开发。尽快启动汕尾中心渔港建设，以汕尾渔港带动全市十大标准化渔港及其商贸配套设施建设，打造海产品物流商贸中心。大力发展海洋捕捞业和水产品深加工业，加快对虾、青蟹、牡蛎、海胆、鲍鱼、海马、海水鱼、淡水鱼、海水网箱、江蓠鱼塭等十大水产养殖基地建设，提高海洋渔业产业化水平，重点抓好恒兴水产等项目上马建设，打造海洋经济支柱产业。

　　努力扩大农民就业。积极开展农业生产技能和"阳光工程"培训，努力把广大农户培养成懂技术、善经营、会管理的现代农业生产者。实施"创业带动就业工程"和"城乡增收扶贫工程"，加快农村富余劳动力转移，增加农民收入，力争年末农民人均收入突破1000美元。扶贫"双到"脱贫户达到80%以上。

　　不断完善农村民主管理。加强基层政权建设，强化对村务公开民主管理工作的指导督促考核，认真总结推广村务公开民主管理的先进经验。提前做好部署，确保完成第5届村委会和第4届社区居委会换届选举任务，推进村、社区居委会规范化建设。

　　八、强力推进财税工作，逐步实现向"发展型财政"转型

　　培植壮大财源。全力推进一批大项目早日投产、发挥效益，提供强劲财源。积极引进科技含量高、辐射能力强、产业链条长的重大生产性项目，培育和发展高利税主导行业和大型企业集团，推动企业上规模、增效益，提升财政收入的质量和稳定性。

　　加强财税征管。充分发挥各级政府以及各职能部门协税护税的积极性，加强对重点税种、重点税源企业和重点项目的管理，管好经济户口，强化纳税评估，争取在税源挖潜堵漏上取得新突破。坚持依法治税，加大税收稽查力度，严厉打击各种偷、逃、骗税行为。认真抓好免抵调以及地方小税种的征收和管理，切实做到应收尽收。

　　稳步推进财税改革。继续深化部门预算改革，提高预算编制水平。配合省完善省以下财政体制和"省直管县"改革，积极做好应对措施，确保改革推进与市、县既得利益的平衡。认真做好对外资企业开征城建税及教育费附加的基础性工作，深入推进税收体制改革，加强部门协作，夯实征管基础，确保开征工作顺利进行。

　　提升财税管理水平。按照确保正常运转和集中财力办大事的原则，优化财政资源配置，压缩一般性行政支出。认真整合财政性引导资金，加大对民生领域和社会事业的支持保障力度，加大对"三农"、科技、教育、卫生、文化、社会保障、保障性住房、节能环保等方面和中小企业、居民消费的支持力度。提高财税收入质量，推进基本公共服务均等化。

　　防范财政和金融风险。依法规范政府投融资行为，完善政府融资平台相关机制。发展规范担保公司和中介机构，构建中小企业融资平台。防范和化解银行业风险，维护财政金融安全。

　　九、强力推进和谐汕尾建设，加快社会事业发展

　　完善社会保障体系。继续抓好社保扩面征缴，巩固和完善社会保险市级统筹，确保养老金等各项社保待遇按时足额发放和收支平衡；扩大新型农村养老保险制度试点，全市249个贫困村优先列入新农保范围，大力实施新农保三年全覆盖计划，即2011年底前覆盖率达到60%，2012年底前实现全覆盖。进一步落实积极就业政策，实施劳动力培训转移和就业援助工程，完善就业服务体系，统筹做好城乡就业服务，保持就业局势稳定；落实"三支一扶"各项优惠政策，加强高校毕业生就业服务工作。及时化解和处理劳资矛盾，构建和谐劳动关系。

　　全力维护社会稳定。进一步完善大平安体系，强化社会治安综合治理，推进平安汕尾建设。继续对治安重点地区和突出问题进行重点整治，依法严厉打击各类违法犯罪活动。做好信访工作，积极探索和建立信访工作新机制，积极预防和妥善处置群体性事件，促进社会和谐稳定。实施"质量强市"战略，开展整顿和规范市场秩序专项行动，加强价格监管和调控，有效防止哄抬物价行为发生，强化产品质量和食品药品安全监管。要按照属地管理原则，进一步落实反走私工作责任制，不断深化反走私综合治理工作。加强安全生产和消防监管，坚决遏制重特大安全事故发生。健全应急机制，提高应对突发事件能力。做好防灾减灾工作。

　　推进各项体制改革。加快行政管理体制改革，减少和规范行政审批，进一步转变政府职能。积极推进富县强镇和简政强镇改革。深化重点领域和关键环节改革，推进财政体制、投融资体制、国有企业改革以及土地管理制度、集体林权制度、海域使用权制度、粮食流通体制和农村流通体制等方面改革。抓好科技、教育、人事、文化、卫生、体育等分类改革。

　　优先发展教育事业。实施"教育扩容工程"，不断完善"议教"制度，落实筹资优先、政策优惠、教师优待、学位优良的"四优"原则，促进义务教育均衡发展。大力发展学前教育、高中阶段教育和中职教育，办好教育园区。重点抓好汕尾科技职业技术学院、华南建筑装饰工程学院、华师附中二期、汕尾理工职业学校、市卫校、综合中专、汕尾职业技术学院改制、北师大陆丰附中、陕西师大海丰附中和四个教育园区续建等项目建设或改制。启动"名校、名校长、名教师"三名工程，切实解决中小学代课教师问题，提高教师工资福利。

　　大力提升文化软实力。积极实施《汕尾市建设文化强市规划纲要（2011-2020年）》，加快推进"文化强市"建设，规划建设一批代表汕尾文化形象的公共文化设施，重点建设中国（汕尾）民间文化艺术馆、市影剧院、文化广场、文化公园等。加强文化遗产的保护和开发利用，支持文化传媒事业产业化，大力发展创意产业、文化消费，提升文化软实力。抓紧谋划筹办好粤东侨博会。

　　大力发展各项社会事业。推进医药卫生事业发展，完善公共卫生和医疗服务体系，强化疾病防控和卫生监督执法。完善体育基础设施，发展体育产业。加大人才引进和培养工作力度。以全省人口计生工作暨信息化建设现场会在我市召开为契机，进一步落实人口和计生工作责任制，推进"转型升类"工程，稳定低生育水平。抓好殡葬改革。发展社会福利和慈善事业。完善救助管理机制。加强国防教育和国家安全教育，做好"双拥"和民兵预备役工作。促进审计、统计、法制、物价、外事侨务、民族宗教、台务、港澳、方志、档案、气象、防震、人防、广播电视、新闻出版、妇女儿童、老龄、残联、红十字会等工作全面发展。

　　十、强力推进政府自身建设，提高政府行政效能

　　加强民主法制建设。自觉接受人大及其常委会的监督，坚持定期向人大及其常委会报告重要工作。主动加强与政协的工作沟通和协商，接受其民主监督。积极听取各民主党派、人民团体和社会各界的意见和建议，支持工会、共青团和妇联等人民团体更好地发挥作用。提高人大议案、政协提案的办理质量和效率。进一步贯彻落实国务院《全面推进依法行政实施纲要》和《国务院关于加强市县政府依法行政的决定》，坚持依法行政、公正司法和严格执法。推进政务公开，巩固和扩大基层民主，深入推进村（居）民自治。加强法制宣传教育，增强公民法律素质。

　　加快转变政府职能。强化经济运行监测和调节，妥善应对各种经济风险。切实改善对市场主体的服务，增强企业投资和社会消费的信心。进一步理顺政府与企业、市场、社会的关系，创新政府管理方式，不断提高政府决策的科学性、民主性和透明度。强化政府的社会管理和公共服务职能，高度关注民生，切实解决涉及群众利益的难点、热点问题，促进基本公共服务均等化，维护社会安定团结。

　　提高政府行政效能。严格按照法定权限和程序行使权力，规范行政行为。以机构改革为契机，创新政府运行机制，理顺部门职责关系。继续用"三大机制"力促工作落实，切实提高政府执行力。加快推进政务公开和电子政务建设，发挥市行政审批电子监察系统的作用，改善投资环境，提高办事效率。加强公务员队伍管理，大力建设服务型机关。强化行政监察、审计等专门监督，实行行政过错责任追究和党政领导干部问责制度。

　　强化政府作风建设。进一步加强对领导干部的廉政教育，认真落实反腐倡廉各项规定，促进政府廉政建设。以提高执行力为主题，深入开展机关作风建设，严格考核，兑现奖罚，营造干事创业的良好氛围。

　　各位代表！回顾"十一五"，我们无比自豪，展望"十二五"，我们信心百倍！2011年工作任务光荣而艰巨，让我们更加紧密地团结在以胡锦涛同志为总书记的党中央周围，在市委的坚强领导下，振奋精神，坚定信心，齐心协力，真抓实干，当好融入珠三角的桥头堡、区域协作发展的生力军，为把汕尾建设成为宜居宜业宜憩的现代化滨海城市而努力奋斗！
